# An Account of Some Experiments with Opium in the Cure of the Venereal Disease

**Published:** 1788

**Authors:** J. F. Coste

**Affiliations:** First Physician to the French Army


LONDON MEDICAL JOURNAL.

### I. *An Account of ſome Experiments with Opium in the Cure of the Venereal Diſeaſe. Extracted from the Correſpondence of the Military Hoſpitals of France, and communicated to Dr.* Simmons *by* J. F. Coſte, *M. D. Firſt Phyſician to the French Army.*

OF trials made with opium, in the cure of the venereal diſeaſe, we have already had occaſion at different times* to give an account. The paper now communicated by Dr. Coſte relates to ſome experiments made on this ſubject, in the year 1785, in the Military Hoſpital at Liſle, in Flanders. Theſe trials were conducted by M. Merlin, one of the phyſicians of the hoſpital, under the immediate inſpection of a Committee appointed for this purpoſe, and which conſiſted of M. Deſmilleville, firſt phyſician of the hoſpital; M. Boucher and M. Sal-

* See Vol. III. page 222; Vol. IV. page 419; and Vol. VI. page 5.

mon, mon, phyficians at Lifle; Meffieurs Chaftanet, father and fon, furgeons of the hofpital; and Meffieurs Guerin and Gigot, furgeons of two regiments in garrifon at Lifle.

The number of patients fet apart for thefe experiments was thirty, and they were felected by the Committee from a much larger number of venereal patients among the troops in garrifon at Lifle. In nine of thofe cafes recourfe had been had to mercury; but either no good effect had been obtained from it, or the relief it afforded had been merely palliative: in the remaining twenty one no proper means of cure had been tried.

An accurate regifter of each cafe was kept by the Committee, who vifited the patients daily during the whole of the treatment. Of thefe regifters Dr. Cofte obferves, that to have printed them entire would have led to a great number of ufelefs repetitions. He has, therefore, deemed it more eligible to give a general view of the facts and obfervations they contain. To this he has added a table, which exhibits a fuccinct view of each cafe.

In fome of the patients who were felected for thefe experiments the difeafe was recent, and the fymptoms were only the local ones of gonorrhœa gonorrhœa and chancres; but in the greater number there were blotches and ulcers on the ſurface of the body, ulcers in the fauces, ophthalmia, nocturnal pains of the bones, nodes, and other uſual attendants on the confirmed lues venerea.

The treatment commenced on the 20th of Auguſt, 1785, with a purgative medicine, by way of preparation, and the day following with the opium. The patients, in general, began with one grain of pure opium; the day following they took two grains; and in this manner the doſe was daily increaſed, by the addition of a grain, till they got to what the phyſician deemed a ſufficient doſe.—The doſe of each day was given at once, about three hours after dinner.

In ſome of the patients the doſe was extended to thirty grains, but no farther, except in one caſe where it was increaſed to thirty-two grains; and this patient, Dr. Coſte obſerves, took 1706 grains of opium during the courſe of his treatment, which laſted ſix months. In this caſe, however, it was found to have no effect on ſome condylomata, which likewiſe reſiſted different topical applications, mercurial ones excepted, theſe having been ſcrupulouſly

 avoided avoided in all the cafes. It was remarked, that the long-continued ufe of opium in this cafe did not occafion the leaft fenfible inconvenience to the patient, even when he took it in the largeft dofes.

In the greater number of the patients the higheft dofe was twenty grains; and this was feldom continued more than four days fucceffively.

The quantity of opium, on an average, taken by thofe who were deemed cured, was three hundred and fifty grains, and this in the fpace of fix weeks. But in feveral of the cafes the quantity adminiftered during the treatment amounted to eleven and even twelve hundred grains, and in one, as we have feen, to much more. Of thefe, fome were cured; but there were others whofe cure was doubtful, and a few in whom the remedy failed altogether.

When it was thought right, from the figns of amendment in a cafe, to leffen the dofe, it was done gradually, with the fame precaution that had been obferved in increafing it. But when, as fometimes happened, any alarming fymptoms were occafioned by the remedy, its ufe was fufpended for feveral days; and this alone, Dr. Cofte obferves, was, in general, fufficient ficient to remove every appearance of inconvenience from the remedy.

The firft effect of the opium, when given in a moderate dofe, was to leffen pain and the fymptoms of morbid irritability. It was feldom before the dofe was increafed to four or five grains that it produced more fenfible effects. The moft common was more or lefs of a difpofition to fweating, which continued during the greater part of the treatment.

The fweating did not leffen the fecretion of urine: on the contrary, it was obferved, that during the whole courfe of the treatment the quantity of urine was increafed.

In a great number of the patients, during the treatment, eruptions appeared on the fkin, attended with itching.

In three of the cafes a pretty copious falivation was obferved to take place *.

The evacuations by ftool were rather increafed than diminifhed. Several of the patients complained of diarrhœa, and only a very few were troubled with obftinate coftivenefs.

One of the inconveniences produced by the remedy in fome of the cafes was a vomiting—

* See the letter from Dr. Cofte, in page 26.

a great difpofition to fleep took place only in a few; but on the contrary, Dr. Cofte obferves, that a want of fleep was a much more general complaint. The greater number of the patients complained of an increafe of internal heat, and the pulfe at the fame time was quickened, when the remedy was given in large dofes.

Befides thefe effects, others were occafionally obferved to take place, fuch as giddinefs, a kind of intoxication, difagreeable dreams, hiccough, palpitation of the heart, and colicky pains. But thefe effects feldom rendered the ufe of any particular remedy neceffary, as they almoft conftantly ceafed on fufpending the ufe of the opium.

Acids were found ufeful in checking the difpofition to vomit, and in diminifhing the propenfity to fleep; and nothing was found to contribute more efficacioufly to relieve the watchfulnefs, and its confequences, than the ufe of fimple emulfions.

The patients were confined to a low regimen, which confifted chiefly of rice. Their drink was compofed of diluting liquors, given with a view to affift in promoting the difpofition to fweating.

The

The patients were required to continue in bed only when the ſweating rendered it neceſſary.

The treatment began, as we have already had occaſion to obſerve, in the month of Auguſt, and on the 14th of December following the Committee agreed unanimouſly in conſidering ſeven of the patients as cured.

In the month of March, 1786, fourteen of the thirty patients were in the ſame unanimous manner conſidered as cured, as were alſo four others, by a majority of the Committee. Of ſeven the cure was, by a majority, declared doubtful; and four were conſidered as not cured, viz. three by the greater number, and one by the whole of the Committee. The thirtieth patient had been diſcharged, ſoon after his admiſſion, as an improper ſubject, being far advanced in a pulmonary conſumption, of which he died ſoon after.

On the 29th of April, 1786, twenty-four of the patients having been again examined by five members of the Committee, the reſult of their opinions was leſs favourable than before. Eight only were now allowed to be cured, viz. two by a majority, and ſix by the whole of the Committee; the cure of nine was by a majority majority confidered as doubtful; and feven were in a fimilar manner declared to be not cured. Of the remainder of the thirty patients three (two of whom at a former meeting had been confidered as cured) were gone from Lifle with their regiment; two had been fent back on account of the fcurvy; and the fixth, as hath been already mentioned, was dead.

*Table of the Cafes in which Opium was adminiftered.*

[In the cafes diftinguifhed by an afterifm no mercury had been exhibited.]

*1. Bedon, aged 25 years, with fwelling of the inguinal glands, and pains of the limbs.

| | |
|---|---|
| The treatment lafted | 125 days. |
| During which he took | 1062 gr. of opium. |
| The higheft dofe (continued four days) was | 20 grains. |
| Cure doubtful. | |

*2. Levaffeur, aged 31, with warts, phymofis, and ulcers in the throat.

| | |
|---|---|
| The treatment lafted | 147 days. |
| During which he took | 913 gr. of opium. |

The

The higheſt doſe (continued four days) was 20 grains.

Cure doubtful.

*3. Leroi, aged 37, with two chancres, and a bubo in ſuppuration.
The treatment laſted 41 days.
During which he took 458 gr. of opium.
The higheſt doſe (con-
tinued two days) was 20 grains.
Cured.

*4. Gavala, aged 39, with phymoſis, chancres on the prepuce, and ſwelling of the left inguinal glands.

The treatment laſted 39 days.

During which he took 346 gr. of opium.

The higheſt doſe (continued four days) was 18 grains.

Cured.

*5. Grillet, aged 20, with phymoſis, chancres, ſwelling of the inguinal glands, and marks of ſcrophula.

The treatment laſted 163 days.

During which he took 987 gr. of opium.

The higheſt doſe (continued four days) was 20 grains.

Not cured.

6. Lambert, aged 25, with warts on the penis and prepuce.

Had

Had been treated ineffectually with mercury.
The treatment with opium lafted - - 42 days.
During which he took 542 gr. of opium.
The higheft dofe (continued four days) was 20 grains.
Cure doubtful.

*7. Delaunay, aged 30, with two buboes, two chancres on the glans penis, and nocturnal pains of the limbs.
The treatment with opium lafted - - 99 days.
During which he took 1121 gr. of opium.
The higheft dofe (continued five days) was 28 grains.
Cured.

*8. Debailly, aged 20, with gonorrhœa, chordee, chancre, fwelling of the inguinal glands, pains, and blotches on the fkin.
The treatment with opium lafted - - 55 days.
During which he took 658 gr. of opium.
The higheft dofe (continued two days) was 21 grains.
Not cured.

*9. Leroux,

*9. Leroux, aged 23, with gonorrhœa, and two painful buboes.

The treatment with opium lafted - - 55 days.
During which he took 539 gr. of opium.
The higheft dofe (continued four days) was 20 grains.
Cured.

*10. Colomb, aged 25, with fwelling of the inguinal glands, chancres, and nocturnal pains.

The treatment with opium lafted - - 55 days.
During which he took 588 gr. of opium.
The higheft dofe (continued five days) was 20 grains.
Not cured.

11. Lainier, aged 24, with warts about the anus, and chancres on the glans and prepuce. Had taken mercury.

The treatment with opium lafted - - 90 days.
During which he took 580 gr. of opium.
The higheft dofe (continued four days) was 21 grains.
Cure doubtful.

*12. Breuil, aged 25, with a bubo in ſuppuration, chancres on the penis, and nocturnal pains.

The treatment with opium laſted - - 79 days.
During which he took 669 gr. of opium.
The higheſt doſe (continued four days) was 21 grains.
Cure doubtful.

*13. Baſtiane, aged 34, with warts on the glans penis, chancres on the velum palati, exoſtoſis of the os frontis, and nocturnal pains.

The treatment with opium laſted - - 121 days.
During which he took 572 gr. of opium.
The higheſt doſe (continued four days) was 17 grains.
Cure doubtful.

*14. Lamouilliere, aged 18, with warts on the glans and prepuce, and a violent ſenſation of heat in the urethra.

The treatment with opium laſted - - 45 days.
During which he took 545 gr. of opium.

 The

The higheſt doſe (continued four days) was 20 grains.

Cured.

*15. Leſeur, aged 22, with chancres on the glans penis.
The treatment with opium laſted - - 101 days.
During which he took 497 gr. of opium.
The higheſt doſe (continued four days) was 18 grains.
Cured.

16. Mazier, aged 25, with chancres on the glans penis, painful ſwelling of the inguinal glands on one ſide, and nocturnal pains.
Had been cured of a former lues by Keyſer's pills.
The treatment with opium laſted - - 110 days.
During which he took 718 gr. of opium.
The higheſt doſe (continued five days) was 18 grains.
Not cured.

*17. Roché, aged 26, with a bubo in the left groin, and a chancre on the prepuce.
The treatment with opium laſted - - 55 days.

 During

During which he took 553 gr. of opium.
The higheſt doſe (continued eight days) was 20 grains.
Cure doubtful.

18. Chaſſelin, aged 19, with warts on the glans and prepuce, which had been preceded by gonorrhœa.

Had tried mercurial frictions and Keyſer's pills without effect.

The treatment with opium laſted - - 180 days.

During which he took 1706 gr. of opium.

The higheſt doſe (continued two days) was 32 grains.

Not cured.

19. Combet, aged 23, with chancres on the glans and prepuce, ſwelling of the inguinal glands, and ulceration at the poſterior part of the right noſtril.

Had tried mercury without effect.

The treatment with opium laſted - - 115 days.

During which he took 1142 gr. of opium.

The higheſt doſe (continued two days) was 18 grains.

Not cured.

20. Fallaci,

20. Fallaci, aged 26, with violent ophthalmia, acute pains in the arms, painful ſwelling of the inguinal glands, and chancres.

Had tried mercury.

The treatment with opium laſted - - 132 days.

During which he took 449 gr. of opium.

The higheſt doſe (continued two days) was 20 grains.

Not cured.

21. Poyer, aged 26, in a ſtate of maraſmus, with puſtules on the face, back, and extremities, uvula deſtroyed, and ulcers on the velum palati.

Had tried mercurial frictions without effect.

The treatment with opium laſted - - 102 days.

During which he took 367 gr. of opium.

The higheſt doſe (continued ſeven days) was 11 grains.

Cure doubtful.

22. Duchon, aged 30, with chancres, eruption on the ſcrotum, conſtant pain in the limbs, and loſs of his hair.

Had taken mercury.

The

The treatment with opium lafted only 38 days, when it was difcontinued on account of the reduced ftate of the patient, who died foon after of phthifis.

*23. Maubert, aged 26, with a deep chancre on the frænum, and fwelling of the inguinal glands.

The treatment with opium lafted - - 125 days.
During which he took 863 gr. of opium.
The higheft dofe (continued three days) was 24 grains.
Not cured.

24. Bouvier, aged 24, with gonorrhœa, hernia humoralis, and chancres.

Had tried mercurial frictions and fublimate.

The treatment with opium lafted - - 56 days.
During which he took 450 gr. of opium.
The higheft dofe (continued eight days) was 18 grains.
Cured.

*25. Marchal, aged 21, with a bubo in each groin, and a wart on the prepuce.

The treatment with opium lafted - - 70 days.

During

During which he took 859 gr. of opium.
The higheſt doſe (continued eight days) was 20 grains.
Cure doubtful.

26. Chapuy, aged 28, with warts on the glans and prepuce, gonorrhœa of a year's ſtanding, and hernia humoralis.

Had tried mercurial frictions.

The treatment with opium laſted - - 42 days.

During which he took 458 gr. of opium.

The higheſt doſe (continued four days) was 18 grains.

Cured.

*27. Rayer, aged 22, with recent gonorrhœa, phymoſis, and chancres on the glans and prepuce.

The treatment with opium laſted - - 83 days.

During which he took 446 gr. of opium.

The higheſt doſe (continued four days) was 15 grains.

Cured.

*28. Rineſſi, aged 22, with gonorrhœa, and hernia humoralis.

The treatment with opium laſted - - 29 days.

During

During which he took 169 gr. of opium.
The higheft dofe (continued four days) was 13 grains.
Cured.

*29. Lecomte, aged 20, with gonorrhœa, phymofis, fwelling of the inguinal glands of the right fide, and pains of the limbs.
The treatment with opium lafted - - 36 days.
During which he took 307 gr. of opium.
The higheft dofe (continued four days) was 15 grains.
Cure doubtful.

*30. Duthuit, aged 23, with bubo, and with chancres on the glans and prepuce.
The treatment with opium lafted - - 106 days.
During which he took 867 gr. of opium.
The higheft dofe (continued 15 days) was 18 grains.
Cure doubtful.

Befides the ten cafes marked *cured* in the foregoing table, there are five others, viz. the 11th, 12th, 21ft, 29th, and 30th, which Dr. Cofte thinks might not improperly be added to the the lift of cures, they having been confidered as cured at a full meeting of the Committee in March, and fome appearances which induced three, out of five, of the members who met the month following, to deem the cures doubtful, not having been fufficient in his opinion to invalidate the firft decifion of the Committee. Dr. Cofte, however, candidly acknowledges that, upon the whole, thefe experiments are far from proving that opium is a fpecific for the lues venerea; but he is of opinion that they afford abundant and indifputable proofs of the ufeful effects we may expect from it in cafes where too great a degree of irritability requires us to fufpend the ufe of mercury, or to moderate the effects of that remedy. He obferves alfo, that it may be advantageoufly applied in a variety of cafes in which mercury has been tried without fuccefs, particularly in thofe in which the fymptoms appear to be as much the effect of an improper ufe of mercury as of any remains of the venereal virus *.

Since the preceding account was communicated to us, Dr. Cofte, in a letter, of which

* This agrees with the obfervations of Mr. Grant on this fubject. See Vol. VI. page 15. — EDITOR.

we ſhall here give an extract, has had the goodneſs to reply to ſome inquiries made by the Editor relative to theſe experiments.

"The cures * I have mentioned," he ſays, "were certainly deemed ſuch on the cleareſt "evidence, and I have the ſatisfaction of be-"ing able to add that they have been perma-"nent.

"Of the three patients you inquire after, "who ſuffered a pretty copious ſalivation, the "firſt had never undergone any mercurial "treatment. He was both ſcorbutic and ſcro-"phulous, and received but little relief from "the uſe of opium. The ſecond had juſt be-"gun the uſe of ſome mercurial remedies "(but of which I am unable to give you either "the forms or the doſes) when he was placed "under the care of Dr. Merlin, who cured "him perfectly with opium. The third, who "was likewiſe ſucceſsfully treated with opium,

* To ſome of the caſes marked in the table as cured, it may perhaps be objected, that as the complaints in thoſe caſes were merely local, they can hardly be conſidered as proofs of the efficacy of opium in the cure of the venereal diſeaſe; eſpecially when we take into the account the uſe of topical applications, the regimen of the patients, the length of time employed in the cure, and other circumſtances.—EDITOR.

"had

" had been cured of a former venereal com-
" plaint, fix years before, by taking Keyfer's
" pills.

" The papers from which I drew up my re-

" port of the experiments do not determine

" fo precifely, as you appear to defire, the

" exact ftate of the pulfe: you may reft af-

" fured, however, that in all the cafes at Lifle

" there was a very fenfible acceleration of the

" pulfe during the action of the opium.

" You feem to wifh that I had defcribed
" more at length the particulars of the feveral
" experiments. But in the extract I was to
" give I thought it beft to confine myfelf to
" the more general circumftances that attended
" them. The complete hiftory, however, of
" all the cafes fhall be given as foon as poffi-
" ble, together with the additional facts which
" the daily practice of our hofpitals continues
" to afford.

" *Verfailles,*

" Nov. 30th, 1787."

